# Identification of Three (Iso)flavonoid Glucosyltransferases From *Pueraria lobata*

**DOI:** 10.3389/fpls.2019.00028

**Published:** 2019-01-25

**Authors:** Xin Wang, Changfu Li, Zilin Zhou, Yansheng Zhang

**Affiliations:** ^1^CAS Key Laboratory of Plant Germplasm Enhancement and Specialty Agriculture, Chinese Academy of Sciences, Wuhan, China; ^2^Shanghai Key Laboratory of Bio-Energy Crops, Research Center for Natural Products, School of Life Sciences, Shanghai University, Shanghai, China; ^3^University of Chinese Academy of Sciences, Beijing, China

**Keywords:** glucosyltransferase, *Pueraria lobata*, flavonoids, isoflavonoids, daidzin

## Abstract

(Iso)flavonoids are one of the largest groups of natural phenolic products conferring great value to the health of plants and humans. *Pueraria lobata*, a legume, has long been used in Chinese traditional medicine. (Iso)flavonoids mainly present as glycosyl-conjugates and accumulate in *P. lobata* roots. However, the molecular mechanism underlying the glycosylation processes in (iso)flavonoid biosynthesis are not fully understood. In the current study, three novel UDP-glycosyltransferases (PlUGT4, PlUGT15, and PlUGT57) were identified in *P. lobata* from RNA-seq data. Biochemical assays of these three recombinant PlUGTs showed all of them were able to glycosylate isoflavones (genistein and daidzein) at the 7-hydroxyl position *in vitro*. In comparison with the strict substrate specificity for PlUGT15 and PlUGT57, PlUGT4 displayed utilization of a broad range of sugar acceptors. Particularly, PlUGT15 exhibited a much higher catalytic efficiency toward isoflavones (genistein and daidzein) than any other identified 7-*O*-UGT from *P. lobata*. Moreover, the transcriptional expression patterns of these *PlUGTs* correlated with the accumulation of isoflavone glucosides in MeJA-treated *P. lobata*, suggesting their possible *in vivo* roles in the glycosylation process.

## Introduction

Flavonoids represent one of the largest classes of polyphenolic secondary metabolites in higher plants (Tohge et al., 2017). Being a branch of flavonoids, isoflavonoids are mostly synthesized in legumes (Veitch, 2009; Veitch, 2013) and show pharmaceutical value and biological activities, e.g., decreasing the risk of cardiovascular diseases and slowing down the aging process (Panche et al., 2016). These compounds also play vital roles in protecting plants against herbivorous attacks and environmental stresses (Dixon and Pasinetti, 2010; Peng et al., 2017). *Pueraria lobata*, a member of the legume family, has been recently studied due to its ability to biosynthesize important isoflavonoids such as puerarin and daidzin (Wong et al., 2011).

Glycosylation is an important process in the downstream steps of isoflavone biosynthesis (Szeja et al., 2017). It is well accepted that glycosylation is mostly catalyzed by plant family 1 UDP-glycosyltransferases (UGTs), which transfer glycosyl moieties from UDP-sugar donors to a wide range of (iso)flavonoid acceptors (Yonekura-Sakakibara, 2009; Egorova and Toukach, 2017). Until now, many UGT genes involved in the (iso)flavonoid metabolism have been identified and characterized based on genome-wide and/or transcriptome sequencing data from plants (Yonekura-Sakakibara and Hanada, 2011; Wu et al., 2017; Yin et al., 2017a,b; Zhao et al., 2017a,b; Liu et al., 2018). For instance, there are 212 putative UGT genes identified from the *Glycine max* genome database, among which several *GmUGTs* involved in isoflavonoid biosynthesis have been functionally characterized *in vivo* and/or *in vitro* (Noguchi et al., 2007; Funaki et al., 2015; Yin et al., 2017b). In addition, some of the UGTs from *Medicago truncatula* (UGT85H2 and UGT78G1) and Black (medicinal) soybean (UGT78K1) have been reported to show activities toward flavonol and flavone substrates, such as kaempferol, quercetin, and apigenin (Modolo et al., 2007; Kovinich et al., 2010).

*Pueraria lobata* roots predominantly accumulate abundant isoflavonoid glycosides and their associated derivatives, among which daidzin and puerarin are two principle glycosides that show various pharmaceutical activities for humans (Rong et al., 1998). For instance, daidzin (daidzein 7-*O*-glycoside) was proven to exhibit antioxidant (Ruiz-Larrea et al., 1997; Heim et al., 2002), anti-atherosclerotic (Lichtenstein, 1998), and antidipsotropic activities (Keung and Vallee, 1993, 1998; Keung et al., 1996). Despite their important values, the mechanisms underlying the glycosylation of (iso)flavonoids in the biosynthetic pathways are still poorly understood. In total, more than one hundred UGT genes have been discovered from *P. lobata* (He et al., 2011; Wang et al., 2015); however, only a few *P. lobata* UGTs have been functionally studied (He et al., 2011; Li et al., 2014; Wang et al., 2015, 2017). In our continuing efforts to investigate glycosylation in the biosynthesis of *P. lobata* isoflavonoids, here we reported three novel isoflavone 7-*O*-UGTs (PlUGT4, PlUGT15, and PlUGT57) from *P. lobata*. The importance of these three PlUGTs in the biosynthesis of *P. lobata* isoflavonoid glycosides was studied by examining the correlation of their gene expressions to the accumulation of the target metabolites.

## Materials and Methods

### Plant Materials and Chemicals

*Pueraria lobata* materials (roots and leaves) were harvested from the plants grown wildly in the open field of Wuhan botanical garden (China), frozen in liquid nitrogen and then stored at -80°C until use. For the methyl jasmonate (MeJA) treatment of *P. lobata* seedlings, *P. lobata* seeds were surface sterilized by 2 min-immersion in 75% ethanol followed by three further washes in sterile distilled water. The sterilized seeds were then germinated on 1/2 MS agar plates in a growth chamber at 25°C with a 16 h/8 h day/night cycle. After 2 weeks, the seedlings with similar height were selected, and transferred into the 1/2 MS liquid medium and grown for another 2 days before treatment. For the treatment, 100 μM MeJA (final concentration) or mock (0.001% ethanol) solutions was added to the liquid medium. Treatment was carried out for 7 days, and the treated seedlings were harvested, flash frozen in liquid nitrogen, and stored at -80°C for the further analysis.

### Cloning and Heterologous Expression of PlUGTs

The open reading frames (ORFs) of *PlUGT4, PlUGT15*, and *PlUGT57* were amplified with the primers listed in Supplementary Table S1, cloned into pGEX-2T vector (GE Healthcare) to give an in-frame C-terminal fusion with a glutathione-S-transferase (GST) tag. These vectors were sequenced for confirmation, and then transformed into *Escherichia coli* BL21 strains for protein expression. The recombinant strains were cultured in LB medium with the addition of 0.5 mM isopropyl-β-D-thiogalactoside (IPTG) to the medium for inducing the expression of the *PlUGTs*. After 20 h of induction at 16°C, the recombinant PlUGTs were extracted and purified using a Glutathione Sepharose 4B kit (GE Healthcare^1^) according to the manufacturer’s manual. In brief, the cell pellets were resuspended in a lysis buffer (0.1 M potassium phosphate pH 8.0, 0.5 M NaCl, 1 mM EDTA, 1 mM dithiothreitol), and disrupted by ultra-sonication on ice. After centrifugation of the cell lysates at 15,000 *g* for 15 min at 4°C, the crude protein extracts were loaded onto a column packed with GST-binding resin. The recombinant PlUGTs were then eluted with elution buffer (0.1 M potassium phosphate pH 8.0, 10 mM reduced glutathione), and desalted into 50 mM Tris–HCl buffer (pH 8.0) by use of a 30 kDa cut-off centrifugal filter. The purity of the recombinant proteins was examined by SDS-PAGE, and further quantified using Bradford assays.

### *In vitro* Enzyme Assay

The enzymatic activity assays were conducted in a 100 μL reaction mixture consisting of 50 mM Tris–HCl buffer (pH 8.0), 10 μg of the recombinant PlUGTs, 5 mM UDP-glucose, and 500 μM acceptor substrates (including phloretin, liquiritigenin, naringenin, eriodictyol, chrysin, luteolin, kaempferol, quercetin, daidzein, genistein, and formononetin). The reactions were carried out at 30°C for 5–30 min and then stopped by adding 100 μL of methanol. The reaction products were subjected to HPLC or LC-MS/MS analysis as described previously (Wang et al., 2016). To determine the kinetic parameters with the substrates of daidzein and genistein, the substrate concentrations in a range from 0 to 500 μM were applied, and the *V*_max_ and *K_m_* values were calculated by a linear regression analysis.

### Phylogenetic Analysis

The amino acid sequences of PlUGTs (PlUGT4, PlUGT15, and PlUGT57) were aligned with previously characterized family 1 UGTs from higher plants by means of Clustal W algorithm (Thompson et al., 2002). A phylogenetic tree was constructed by neighbor-joining method using MEGA 7.0 software (Kumar et al., 2016). The nodes were evaluated with 1000 bootstrap repeats. The accession numbers of the UGTs used for the phylogenetic analysis are listed in Supplementary Table S2.

### Quantitative Real-Time Reverse Transcription PCR (qRT-PCR)

Total RNA was isolated from samples using an EASYspin plant RNA extraction kit (Aidlab Biotechnologies, Co., Ltd., China) according to the provided protocol. RNA was treated with DNase I (Thermo Fisher Scientific, United States) to remove any genomic DNA contamination, and reverse transcribed to first-strand cDNA using the M-MLV reverse transcriptase (Thermo Fisher Scientific, United States). qRT-PCR was carried out with three biological replicates using SYBR Green Master Mix (Roche, Mannheim, Germany). The PCR program was set as follows: 95°C for 10 min, then 40 cycles of 95°C for 30 s, 55°C for 1 min. The *P. lobata* actin gene (accession number HO708075) was used as an internal standard. All the primers used in qRT-PCR are listed in Supplementary Table S1.

### Metabolites Extraction From the *P. lobata* Seedlings

The plant materials were ground to a fine powder in liquid nitrogen with a mortar and the powdered samples were dried at 50°C for 48 h. Ten milligrams of dry weight samples were extracted with 1 mL of methanol by sonication in a water bath for 20 min. The extracts were centrifuged at 12,000 rpm for 15 min and the supernatant was transferred to a new tube. The pelleted sample debris was re-extracted following the procedure above for a total of three extractions. The supernatants from the same samples were combined and dried by using a rotary evaporator at 40°C, re-dissolved in 500 μL of methanol, and filtered through a 0.45 μm filter syringe prior to HPLC analysis. The HPLC and LC-MS analysis were performed as described previously (Li et al., 2016).

## Results

### Identification of the Putative (Iso)flavonoid PlUGTs

**FIGURE 1:**
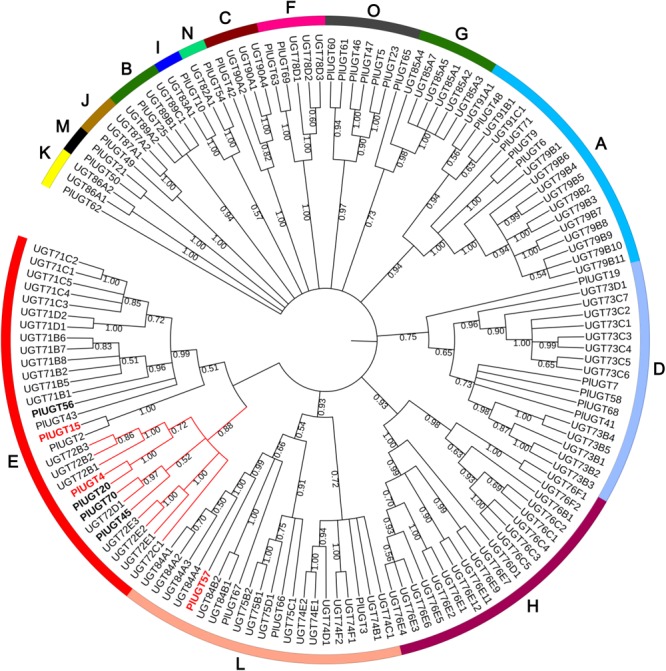
Phylogenetic tree of UDP-sugar-glucosyltransferases (UGTs) from *Pueraria lobata* and *Arabidopsis thaliana*. The neighbor-joining tree was constructed by analysis of the 38 full-length PlUGTs and a set of AtUGTs using MEGA 7.0 software with 1000 bootstrap replicates. The protein names and accession numbers are listed in the Supplementary Table S2.

In a previous study, we performed RNA-seq using *P. lobata* leaves and roots (Wang et al., 2015). Using a BLAST search, a total of 117 putative PlUGTs were identified with 38 PlUGTs bearing complete coding regions in the transcriptome database. A phylogenetic analysis of the 38 full-length PlUGTs was performed with a set of *Arabidopsis thaliana* UGTs (AtUGTs), showing that they were scattered into 14 different groups (Figure 1). In addition to PlUGT2, an (iso)flavone 4′,7-*O*-diglucoside UGT identified in our previous study (Wang et al., 2016), four PlUGTs (PlUGT4, PlUGT15, PlUGT20, PlUGT45) with high expression levels in the root (RPKM values >2 in the root, Supplementary Figure S1 and Supplementary Table S3) were found in the UGT72 subgroup, many members of which were reported to be involved in flavonoid biosynthesis (Yin et al., 2017a,b). As the UGT71B and UGT84A members exhibited a very close relationship to UGT72, PlUGTs in these two subgroups were also selected as the candidates. Besides PlUGT43, an isoflavonoid 8-*C*-UGT identified previously (Wang et al., 2017), two PlUGTs (PlUGT56 and PlUGT57) were found in the UGT71B and UGT84A, respectively. Since PlUGT56 had a low transcript level in the root (Supplementary Figure S1 and Supplementary Table S3), it was not further analyzed. Therefore, the five PlUGTs (PlUGT4, PlUGT15, PlUGT20, PlUGT45, and PlUGT57) were considered as the candidates in this study. However, only three full-length *PlUGTs* (PlUGT4, PlUGT15, and PlUGT57) were successfully amplified from *P. lobata* roots when we performed RT-PCR. The nucleotide sequences of the three PlUGTs were submitted to NCBI database with accession number of MG598529, KU311041, and MG598530 (Supplementary Table S2). The open reading frames of the three *PlUGTs* (PlUGT4, PlUGT15, and PlUGT57) ranged from 1,398 to 1,434 bp with deduced amino acids lengths of 464–477. According to the UGT Nomenclature Committee^2^, these three PlUGTs were designated as UGT72Y3 (PlUGT4), UGT88E23 (PlUGT15), and UGT84F7 (PlUGT57), respectively. Multiple alignment of these PlUGTs with several other characterized (iso)flavonoid UGTs showed that they shared a relative high sequence identity, and all possessed a plant secondary product glycosyltransferase (PSPG) consensus sequence at the C-terminus (Supplementary Figure S2).

**FIGURE 2:**
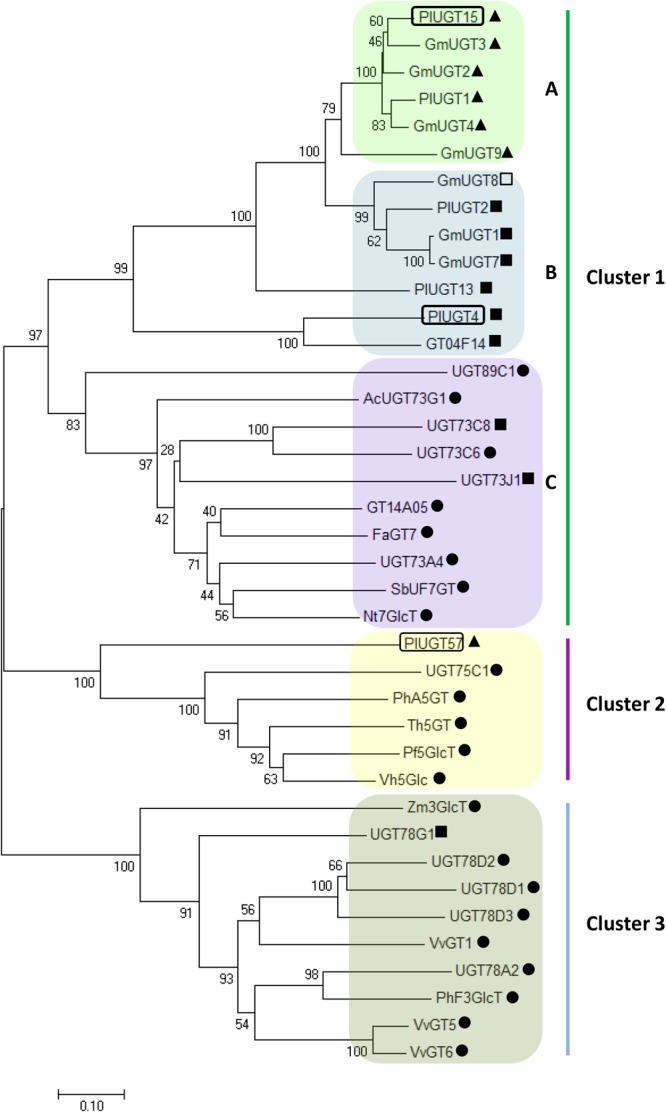
Phylogenetic tree of *Pueraria lobata* UDP-sugar-glucosyltransferases (UGTs) together with previously characterized plant UGTs. The deduced amino acid sequences were aligned by Clustal W and a Neighbor-Joining tree was constructed using MEGA 7.0 software with 1000 bootstrap replicates. The evolutionary distances were computed using the Poisson correction method and the bar indicates 0.1 amino acid substitutions per site. The accession numbers of these UGTs are listed in Supplementary Table S2. The UGTs in the tree were classified into three clusters based on their glycosylation activity at different positions of (iso)flavonoid substrates. Cluster 1 represents the 7-*O*-UGTs that were able to transfer sugar donors to the 7-OH position of (iso)flavone aglycones. Cluster 2 includes the 5-*O*-UGTs, and Cluster 3 indicates the 3-*O*-UGTs. ▲ indicates isoflavonoid UGTs; • refers to flavonoid UGTs; ■; represents the UGTs that can use both flavonoid and isoflavonoid substrates; □ no activity toward flavonoid or isoflavonoid.

To further study the potential functions of these PlUGTs, the three PlUGTs (PlUGT4, PlUGT15, and PlUGT57) were subjected to a phylogenetic analysis together with well-characterized plant UGTs (Figure 2). The PlUGTs were grouped into two of the three clusters (Cluster 1: 7-*O*-UGT, Cluster 2: 5-*O*-UGT, and Cluster 3: 3-*O*-UGT). The clusters in the phylogenetic tree are divided by the *in vitro* glycosyltransferase activity on the different positions of (iso)flavonoid substrates. PlUGT4 and PlUGT15 were clustered into the same group with several plant 7-*O*-UGTs (Cluster 1), while PlUGT57 showed a relatively closer relationship to 5-*O*-UGTs (Cluster 2). Moreover, PlUGT4 and PlUGT15 were separately scattered into two of three subgroups in Cluster 1. In subgroup A, PlUGT15 exhibited relatively higher homolog to the isoflavone specific 7-*O*-UGTs, including four soybean UGTs (GmUGT1, GmUGT2, GmUGT3, and GmUGT4) and PlUGT1. In subgroup B, PlUGT4 clustered into same branch as three GmUGTs and three PlUGTs (PlUGT2, PlUGT13, GT04F14), which display 7-*O*-glucosylation activities both to isoflavone and flavone substrates (He et al., 2011; Li et al., 2014; Wang et al., 2016).

### Biochemical Characterization of PlUGT4, PlUGT15, and PlUGT57

**FIGURE 3:**
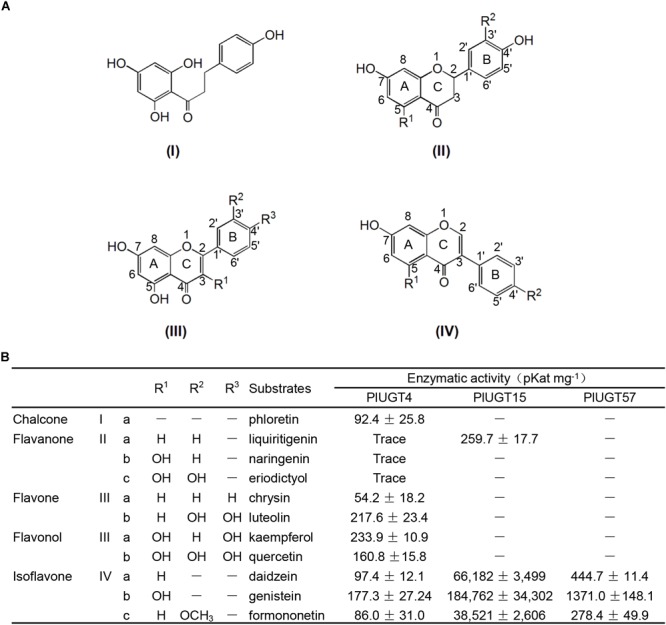
Enzyme activities of three recombinant PlUGTs with different (iso)flavonoid substrates. **(A)** Chemical structures of (iso)flavonol substrates; **(B)** enzyme activities of the recombinant PlUGT4, PlUGT15, and PlUGT57 with 11 (iso)flavonol aglycones as acceptor substrates and UDP-glucose as the donor substrate. Values represent the means ± SD from three replicate assays.

**FIGURE 4:**
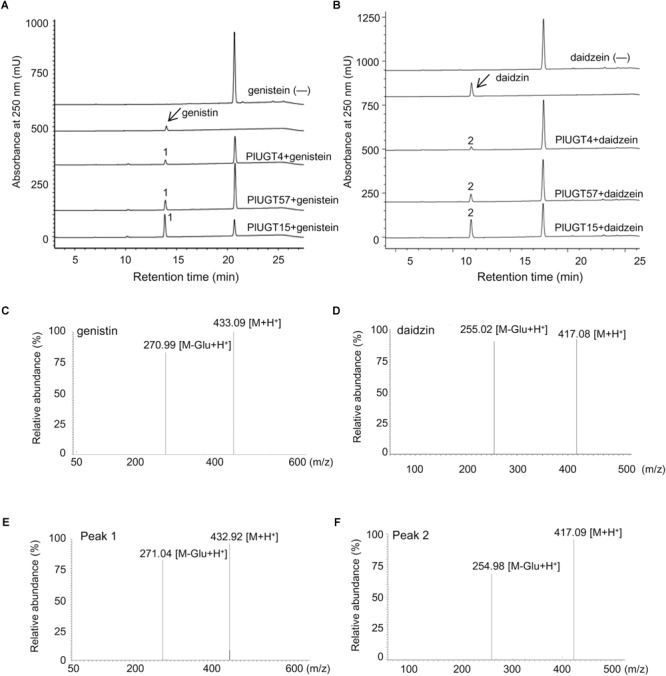
Analysis of the enzymatic reaction products of PlUGTs with isoflavonoid substrates. HPLC chromatograms of reaction products of PlUGT4/PlUGT15/PlUGT57 with genistein **(A)** and daidzein **(B)** substrates. Mass fragmentation pattern of genistin (genistein 7-*O*-glucoside, **C**) and daidzin (daidzein 7-*O*-glucoside, **D**) standard. **(E,F)** are the mass fragmentation pattern of Peak 1 and peak 2, repetitively. (—) Indicate control assays without PlUGT.

Based on the results mentioned above that *PlUGT4, PlUGT15*, and *PlUGT57* were identified as the candidates potentially involved in the biosynthesis of *P. lobata* isoflavone glycosides, we tested their activities with different substrates, whose structures are shown in the Figure 3A. Each PlUGT was expressed in *E. coli* and further purified from the bacterial protein extracts via GST affinity chromatography (Supplementary Figure S3). Using UDP-glucose as the sugar donor, all three PlUGTs converted genistein, daidzein, and formononetin to their corresponding 7-*O*-glucosides (Figure 4 and Supplementary Figure S4), suggesting that they are 7-*O*-UGTs. The substrate specificities of the three PlUGTs showed that PlUGT15 and PlUGT57 accepted isoflavone aglycones while having little to no recognition of the other types of substrates (Figure 3B and Supplementary Figure S4). In contrast, the PlUGT4 protein accepted both flavone and isoflavone substrates with flavones being the better substrates for this enzyme (Figure 3B and Supplementary Figure S5). The best substrate for the PlUGT15 and PlUGT57 was genistein, followed by daidzein and formononetin. It should be mentioned that the specific 7-*O*- activities of PlUGT15 toward the isoflavones were more than 100 and 1000 times higher than those of PlUGT57 and PlUGT4, respectively (Figure 3B), suggesting only PlUGT15 might be the true isoflavone 7-*O*-UGT *in vivo*. When kaempferol and quercetin were used as the acceptors for PlUGT4, multiple products were detected, indicating that they were glucosylated at different positions by this enzyme (Supplementary Figure S5). Next, the kinetic parameters of these three PlUGTs toward daidzein and genistein were measured with UDP-glucose as the sugar donor (Supplementary Figure S6). When daidzein was used as the acceptor, the *K_m_* values of PlUGT4, PlUGT15, and PlUGT57 were 110.8 ± 36.4 μM, 29.5 ± 5.9 μM, and 16.3 ± 1.87 μM, respectively. The *K_m_* values when genistein was applied as the substrate were relatively lower. The *K_cat_*/*K_m_* values of PlUGT15 for either daidzein or genistein were about 50- to 5000-fold higher than those of PlUGT4 and PlUGT57 for the two acceptors (Table 1).

**Table 1:** Kinetic parameters of three recombinant *Pueraria lobata* UDP-sugar-glucosyltransferases (PlUGT) toward isoflavonoid aglycones.

UGTs	Substrates	K_m_ (μM)	K_cat_ (S^-1^)	K_cat_/K_m_ (M^-1^s^-1^)
P1UGT4	Daidzein	110.8 ± 36.4	0.0075 ± 0.0009	67.1
	Genistein	87.3 ± 38.9	0.014 ± 0.0021	1.56 × 10^2^
P1UGT15	Daidzein	29.5 ± 5.87	5.18 ± 0.27	1.75 × 10^5^
	Genistein	13.8 ± 7.09	10.7 ± 2.68	7.75 × 10^5^
P1UGT57	Daidzein	16.3 ± 1.87	0.034 ± 0.0009	2.10 × 10^3^
	Genistein	7.68 ± 3.58	0.11 ± 0.01	1.37 × 10^4^

*Daidzein and genistein were used as sugar acceptors, and UDP-glucose as the donor. Results are means ± SD of triplicate enzymatic assays.*

### Effects of MeJA Treatments on the Expression of the *PlUGTs* and Accumulation of Isoflavones

**FIGURE 5:**
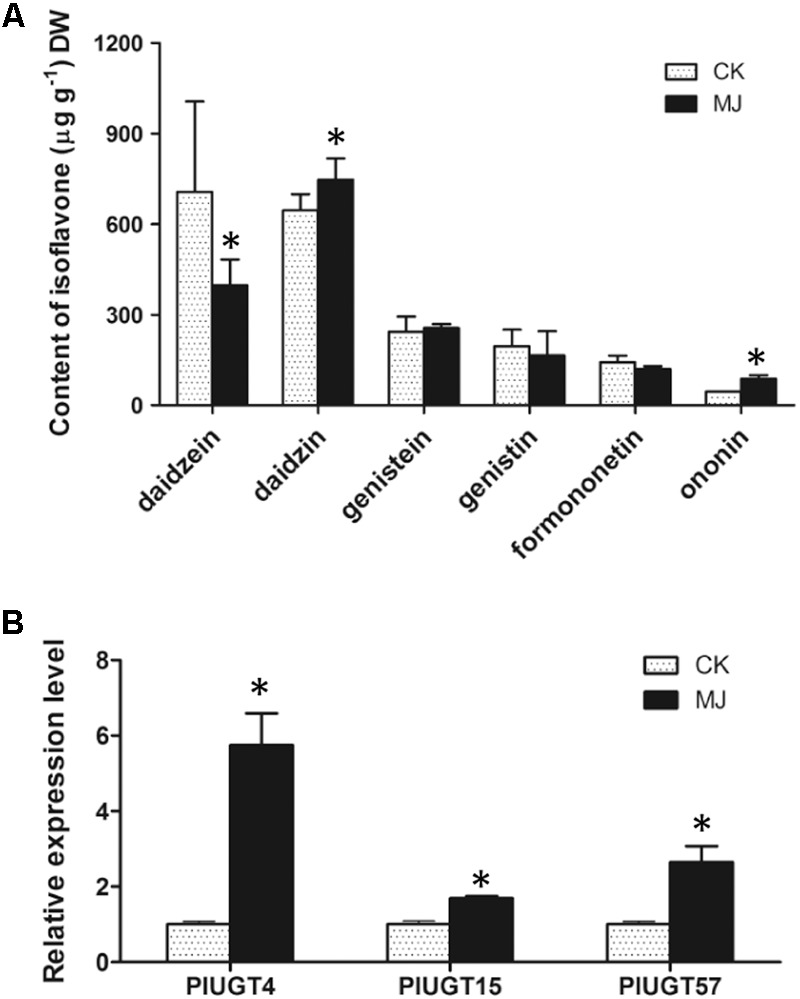
The isoflavone contents and the expression levels of *PlUGTs* in *Pueraria lobata*. Two weeks old seedlings with similar growth height were selected for treating with 100 μM (final concentration) of methyl jasmonate (MJ) for 7 days. CK indicates the mock control plants that were treated with the equal amount of menthol (0.001%, v/v) for the same period. **(A)** The isoflavone contents were analyzed by HPLC as described in the Section “Materials and Methods.” **(B)** The expression levels of *PlUGTs* were monitored by qRT-PCR using the actin gene as the reference standard. Data expressed as means ± SD from three to five biological replicates. ^∗^Indicates there is a significant difference between MeJA-treated plants and mock controls (*p* < 0.05).

It has been reported that MeJA is a critical elicitor in regulating the biosynthesis of plant isoflavonoids (Naoumkina et al., 2007; Farag et al., 2008). In *Pueraria* species, the contents of isoflavone glycosides are generally increased after MeJA elicitation (Goyal and Ramawat, 2008; Korsangruang et al., 2010; Li et al., 2014). To examine whether the expression of UGTs correlated with the isoflavone glucosides biosynthesis, 2 week old *P. lobata* seedlings were subjected to 100 μM MeJA treatment for 7 days, and the contents of daidzein, genistein, and formononetin as well as their corresponding 7-*O*-glucosides (daidzin, genistin, and ononin) were measured. As shown in Figure 5A, the seedlings treated by the MeJA had significantly higher levels of daidzin and ononin compared to their levels in the control plants, while showing a reduction in the contents of daidzein and formononetin, especially that of daidzein which was decreased by 43.5%. No significant changes in the contents of genistein and genistin were observed between the MeJA-treated and control plants. Expression of *PlUGT4, PlUGT15*, and *PlUGT57* were analyzed in the control and MeJA-treated plants using qRT-PCR. Results obtained show that all three *PlUGT* genes were up-regulated compared to the control samples (Figure 5B).

## Discussion

Isoflavones are one of the most abundant flavonoids in legumes (Dixon and Pasinetti, 2010; Szeja et al., 2017). Glycosylated isoflavones, being the major forms of isoflavones in *P. lobata* roots, contribute greatly to plant defense and human health (Veitch, 2013; Zhou et al., 2016). Although several genes involved in the common pathway for isoflavone biosynthesis have been well defined in legumes, the downstream steps for glycosylation are still waiting to be uncovered (Le Roy et al., 2016).

Previously, a total of 117 putative *PlUGT* genes were identified in the root and leaf transcriptome database for *P. lobata* (Wang et al., 2015), which is significantly lower than the 212 putative UGT genes discovered in the *G. max* genome, but close to the number of UGTs (125) in another model legume *Cicer arietinum* (Sharma et al., 2014). In the current study, we have characterized three novel PlUGTs (PlUGT4, PlUGT15, and PlUGT57), and confirmed with *in vitro* biochemical assays that these PlUGTs exhibit activity toward various (iso)flavonoids. Among them, PlUGT15 and PlUGT57 showed strict substrate specificity with highly specific activity toward isoflavone aglycones (daidzein, genistein and formononetin) (Figure 3). Additionally, these two PlUGTs exhibited very strict regio-specificity, and 7-*O*-glucosides were the sole enzymatic products in the *in vitro* reactions. On the other hand, PlUGT4 showed relatively broad activity toward multiple phenolic compounds and was likely to glycosylate substrates at different positions of hydroxyl groups (Figure 3B and Supplementary Figure S5). Other UGTs with broad substrate profiles have also been found in *P. lobata* and other legumes, such as PlUGT2 and GT04F14 from *P. lobata*; GmUGT1 and GmUGT2 from soybean; and UGT72V3 from *Lotus japonicus* (He et al., 2011; Wang et al., 2016; Yin et al., 2017a,b).

From the results of phylogenetic analysis, PlUGT15 and all the other UGT proteins (GmUGT3, GmUGT4, and GmUGT9 from soybean, PlUGT1 from *P. lobata*) in subgroup A (Cluster 1) were experimentally characterized as highly specific isoflavone 7-*O*-UGTs (Li et al., 2014; Funaki et al., 2015), while PlUGT4 and the other UGTs clustered into subgroup B (Cluster 1) displayed broad substrate activities, indicating the phylogenetic clustering of PlUGT4 and PlUGT15 were well matched with their *in vitro* enzymatic assays. However, although PlUGT57 has been proven to be an isoflavone-specific 7-*O*-UGT based on our *in vitro* biochemical evidence, when we looked at the phylogenetic tree, it was found to be grouped into Cluster 2 (5-O-*UGTs*) (Figure 2). Similar results were also reported by other groups. For instance, a CsUGT75L12 from tea plants (*Camellia sinensis*) was predicted to exhibit 5-*O*-glycosyltransferases activity by phylogenetic analysis, but was functionally proven to display 7-*O*-glucosylation activity toward multiple phenolic substrates (Dai et al., 2017). Therefore, the information about the substrate and/or regio-specificity of plant UGTs cannot be predicted only by sequence similarity and phylogenetic analysis (Modolo et al., 2007).

For the enzyme kinetic parameters, all three PlUGTs (PlUGT4, PlUGT15, and PlUGT57) possessed higher affinity toward genistein (with low *K_m_* and high *K_cat_*/*K_m_* values) as compared to daidzein, which was similar to the enzymatic properties for PlUGT1 and PlUGT2 (Li et al., 2014; Wang et al., 2016). However, it should be noted that PlUGT15 exhibited much higher catalytic efficiency values (*K_cat_*/*K_m_*) toward isoflavones than any other identified 7-*O*-UGTs (PlUGT1, PlUGT4, and PlUGT57) from *P. lobata*. For instance, the *K_cat_*/*K_m_* value toward daidzein for PlUGT15 (1.75 × 10^5^ M^-1^s^-1^) is 3.6-fold higher than that for PlUGT1 (3.79 × 10^4^ M^-1^s^-1^), and about 80-fold higher than PlUGT57 (2.10 × 10^3^ M^-1^s^-1^).

To investigate the importance of these three PlUGTs in the glycosylation of *P. lobata* isoflavonoids, we studied the correlation of their gene expression levels and the accumulation of target metabolites using MeJA as the elicitor. It appeared that the gene expression profiles of the three *PlUGTs* positively correlated with the content of daidzin, the principle 7-*O*-isoflavonoid glycoside in *P. lobata* and other legumes, but showed a negative relationship to the level of its corresponding aglycone (daidzein) in the MeJA-treated plants, suggesting their possible roles in the process of (iso)flavonoid biosynthesis *in vivo*. Considering the high catalytic efficiency *in vitro*, PlUGT15 was proposed to be the major isoflavonoid-specific 7-*O*-UGT enzyme in *P. lobata*. However, the full understanding of the role of PlUGTs *in vivo* may be achieved if knock-down or overexpression approaches were performed in *P. lobata* itself. Nevertheless, our findings provide valuable *UGT* gene resources for metabolic engineering of isoflavonoids in legumes and microorganisms in the future.

## Author Contributions

YZ conceived the original research. XW performed the most experiments. CL prepared with the plant materials and assisted with chemical analysis. ZZ performed the gene cloning. XW and YZ wrote the article.

## Conflict of Interest Statement

The authors declare that the research was conducted in the absence of any commercial or financial relationships that could be construed as a potential conflict of interest.

## Footnotes

1 http://www3.gehealthcare.com/

2 http://prime.vetmed.wsu.edu/resources/udp-glucuronsyltransferase-homepage
