# Supplementary material for: Identification of Three (Iso)flavonoid Glucosyltransferases From Pueraria lobata
Source: Front Plant Sci. 2019 Jan 25;10:28. doi: 10.3389/fpls.2019.00028 (PMC6362427; doi:10.3389/fpls.2019.00028)
Supplement: Supplementary file 2 [file Table_2.DOCX]

**Table S2** The accession numbers of plant glycosyltransferases used in this study.

| **Protein Name** | **Species** | **Accession number** |
| --- | --- | --- |
| AcUGT73G1 | *A.cepa* | AY62062 |
| AmC4GT | *Antirrhinum majus* | Q33DV3 |
| FaGT7 | *Fragaria ananassa* | DQ289588 |
| GmUGT1 | *Glycine max* | BAO79433 |
| GmUGT2 | *Glycine max* | AB904891 |
| GmUGT3 | *Glycine max* | AB904892 |
| GmUGT4 | *Glycine max* | AB904893 |
| GmUGT7 | *Glycine max* | AB904894 |
| GmUGT8 | *Glycine max* | AB904895 |
| GmUGT9 | *Glycine max* | AB904896 |
| GT03H24 | *Pueraria lobata* | ADV71363 |
| GT04F14 | *Pueraria lobata* | HQ219042 |
| GT14A05 | *Pueraria lobata* | HQ219047 |
| HpUGT90A7 | *Pilosella officinarum* | ACB56926 |
| LvC4GT | *Linaria vulgaris* | BAE48240 |
| Nt7GlcT | *Nicotiana tabacum* | AF346431 |
| Pf5GlcT | *Perilla frutescens* | Q0WW21 |
| PhA5GT | *Petunia hybrida* | BAA89009.1 |
| PhF3GlcT | *P. hybrida* | AAD55985 |
| PlUGT1 | *Pueraria lobata* | KC473565 |
| PlUGT13 | *Pueraria lobata* | A0A067YBQ3 |
| **PlUGT15** | ***Pueraria lobata*** | **KU311041** |
| PlUGT2 | *Pueraria lobata* | KU311040 |
| **PlUGT4** | ***Pueraria lobata*** | **MG598529** |
| **PlUGT57** | ***Pueraria lobata*** | **MG598530** |
| PlActin | *Pueraria lobata* | HO708075 |
| SbUF7GT | *Scutellaria baicalensis* | AB031274 |
| Th5GT | *Torenia hybrida* | AB076698 |
| UGT73A4 | *B. vulgaris* | AY526080 |
| UGT73C6 | *A. thaliana* | AEC09298 |
| UGT73C8 | *M. truncatula* | DQ875459 |
| UGT73J1 | *Allium cepa* | AY62063 |
| UGT75C1 | *A. thaliana* | AAL69494 |
| UGT78A2 | *Aralia cordata* | AB103471 |
| UGT78D1 | *A. thaliana* | NP_197205 |
| UGT78D2 | *A. thaliana* | NP_197207 |
| UGT78D3 | *A. thaliana* | NP_197205 |
| UGT78G1 | *Medicago truncatula* | A6XNC6 |
| UGT89C1 | *A. thaliana* | Q9LNE6 |
| Vh5GlcT | *Verbena hybrida* | BAA36423 |
| VvGT1 | *Vitis vinifera* | AAB81683 |
| VvGT5 | *Vitis vinifera* | BAI22846 |
| VvGT6 | *V. vinifera* | BAI22847 |
| Zm3GlcT | *Zea mays* | X13501 |
