# Supplementary material for: Identification of Three (Iso)flavonoid Glucosyltransferases From Pueraria lobata
Source: Front Plant Sci. 2019 Jan 25;10:28. doi: 10.3389/fpls.2019.00028 (PMC6362427; doi:10.3389/fpls.2019.00028)
Supplement: Supplementary file 4 [file Image_1.pdf]

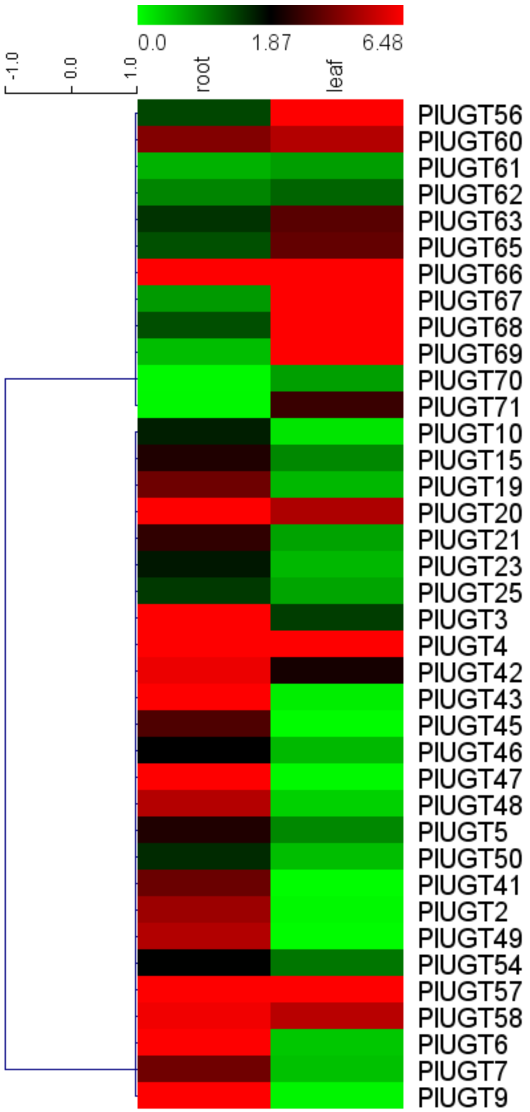

Supplementary Figure S1 Hierarchical cluster analysis of transcript levels of PlUGTs in the *Pueraria lobata* root and leaf. The genes expression data were retrieved from the RNA-seq results (Wang et at, 2015), and the clustering heatmap was created by using the MultiExperiment Viewer (MEV) software.
