# Supplementary material for: Identification of Three (Iso)flavonoid Glucosyltransferases From Pueraria lobata
Source: Front Plant Sci. 2019 Jan 25;10:28. doi: 10.3389/fpls.2019.00028 (PMC6362427; doi:10.3389/fpls.2019.00028)
Supplement: Supplementary file 5 [file Image_2.pdf]

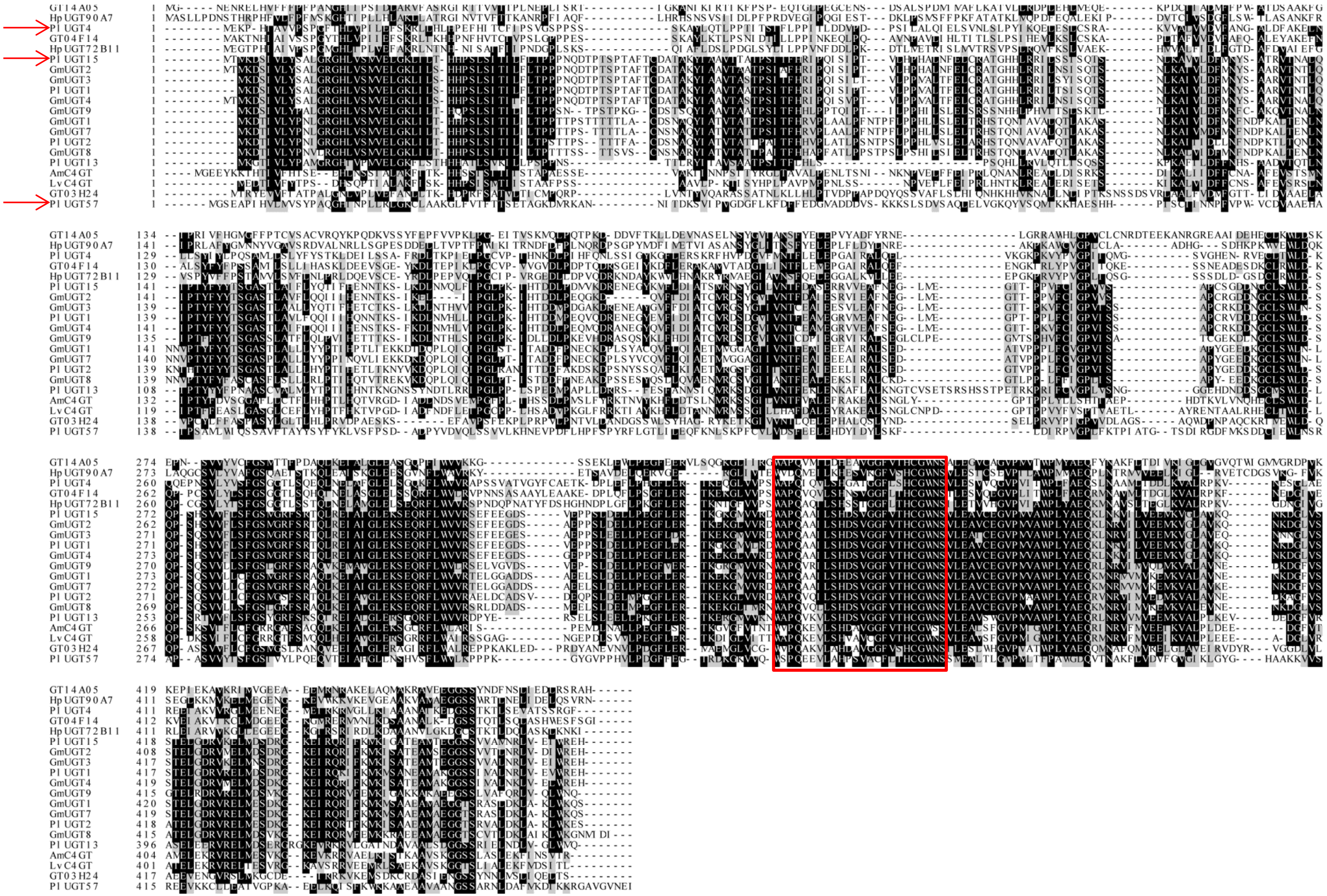

Supplementary Figure S2 Multiple alignment of the sequences of PlUGTs with other functionally characterized UGTs from higher plants. The red arrows indicate PlUGT4, PlUGT15, and PlUGT57 respectively. The rectangle area denotes the plant secondary product glycosyltransferase (PSPG) consensus sequence at the C-terminus.
