# Supplementary material for: Identification of Three (Iso)flavonoid Glucosyltransferases From Pueraria lobata
Source: Front Plant Sci. 2019 Jan 25;10:28. doi: 10.3389/fpls.2019.00028 (PMC6362427; doi:10.3389/fpls.2019.00028)
Supplement: Supplementary file 6 [file Image_3.pdf]

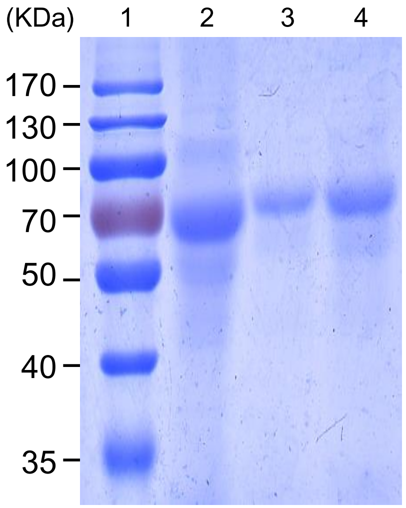

Supplementary Figure S3 SDS-PAGE analysis of the purified recombinant PlUGTs. Lane 1, protein ladder; Lane 2, PlUGT4; Lane 3, PlUGT15; Lane 4, PlUGT57.
