# Supplementary material for: Identification of Three (Iso)flavonoid Glucosyltransferases From Pueraria lobata
Source: Front Plant Sci. 2019 Jan 25;10:28. doi: 10.3389/fpls.2019.00028 (PMC6362427; doi:10.3389/fpls.2019.00028)
Supplement: Supplementary file 7 [file Image_4.pdf]

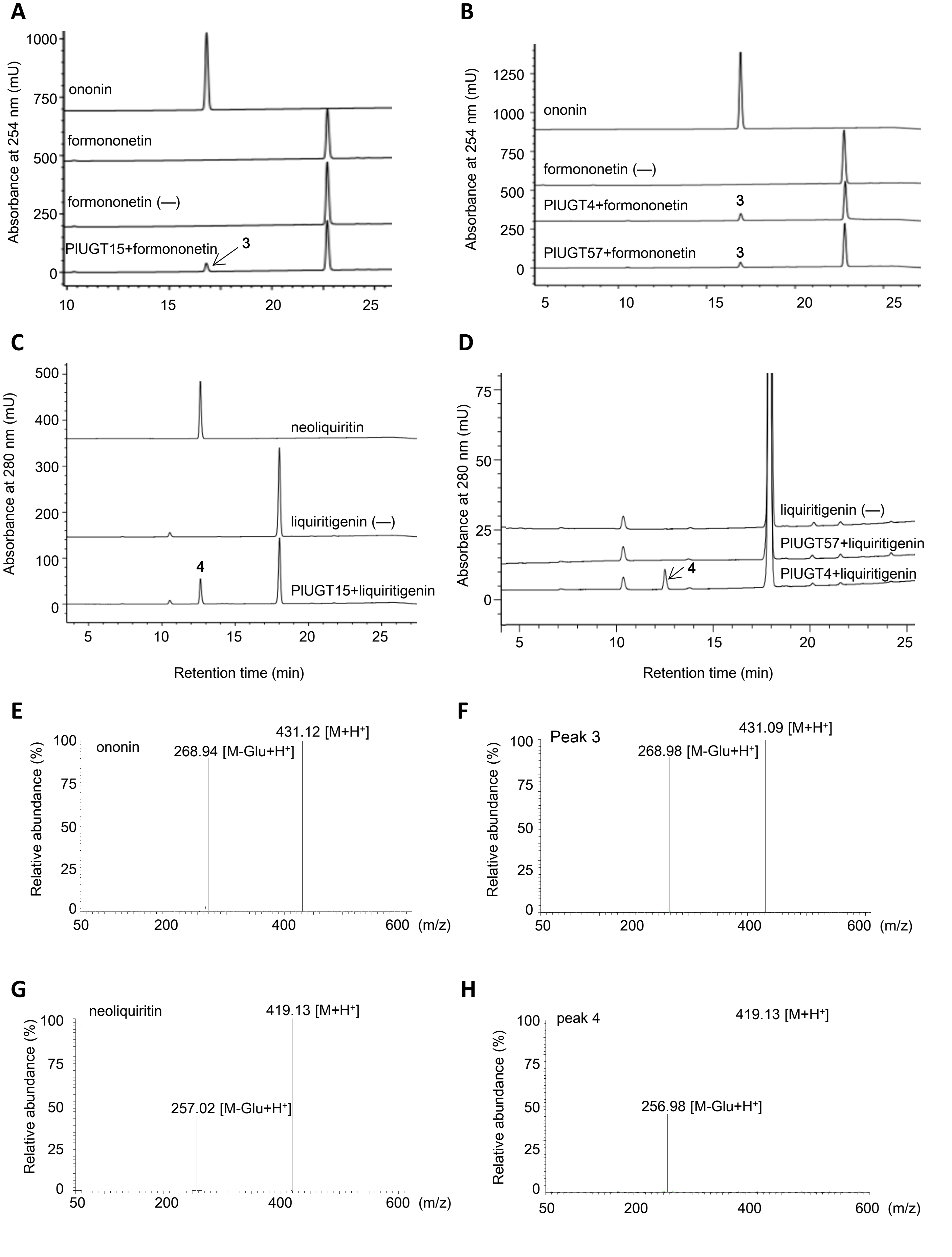
A
B
C
D
E
F
G
H
Absorbance at 254 nm (mU)
ononin
formononetin
formononetin (—)
PlUGT15+formononetin
3
PlUGT4+formononetin
PlUGT57+formononetin
Absorbance at 280 nm (mU)
neoliquiritin
liquiritigenin (—)
PlUGT15+liquiritigenin
4
PlUGT57+liquiritigenin
PlUGT4+liquiritigenin
Retention time (min)
Relative abundance (%)
431.12 [M+H⁺]
268.94 [M-Glu+H⁺]
Peak 3
431.09 [M+H⁺]
268.98 [M-Glu+H⁺]
419.13 [M+H⁺]
257.02 [M-Glu+H⁺]
peak 4
256.98 [M-Glu+H⁺]
(m/z)

Supplementary Figure S4 Analysis of the enzymatic reaction products of three recombinant *Pueraria lobata* UDP-sugar-glucosyltransferases (PlUGT) with (iso)flavonoid aglycones. HPLC chromatograms of reaction products of PlUGT4/PlUGT15/PlUGT57 toward formononetin (A, B) and liquiritigenin (C, D) as acceptor substrates and UDP-glucose as the donor substrate. Mass fragmentation pattern of ononin (formononetin 7-*O*-glucoside, E) and neoliquiritin (liquiritigenin 7-*O*-glucoside, G) standard. (F) and (H) indicates the mass fragmentation pattern of peak 3 and peak 4. (—) indicate control assays without PlUGT protein.
