# Supplementary material for: Identification of Three (Iso)flavonoid Glucosyltransferases From Pueraria lobata
Source: Front Plant Sci. 2019 Jan 25;10:28. doi: 10.3389/fpls.2019.00028 (PMC6362427; doi:10.3389/fpls.2019.00028)
Supplement: Supplementary file 8 [file Image_5.pdf]

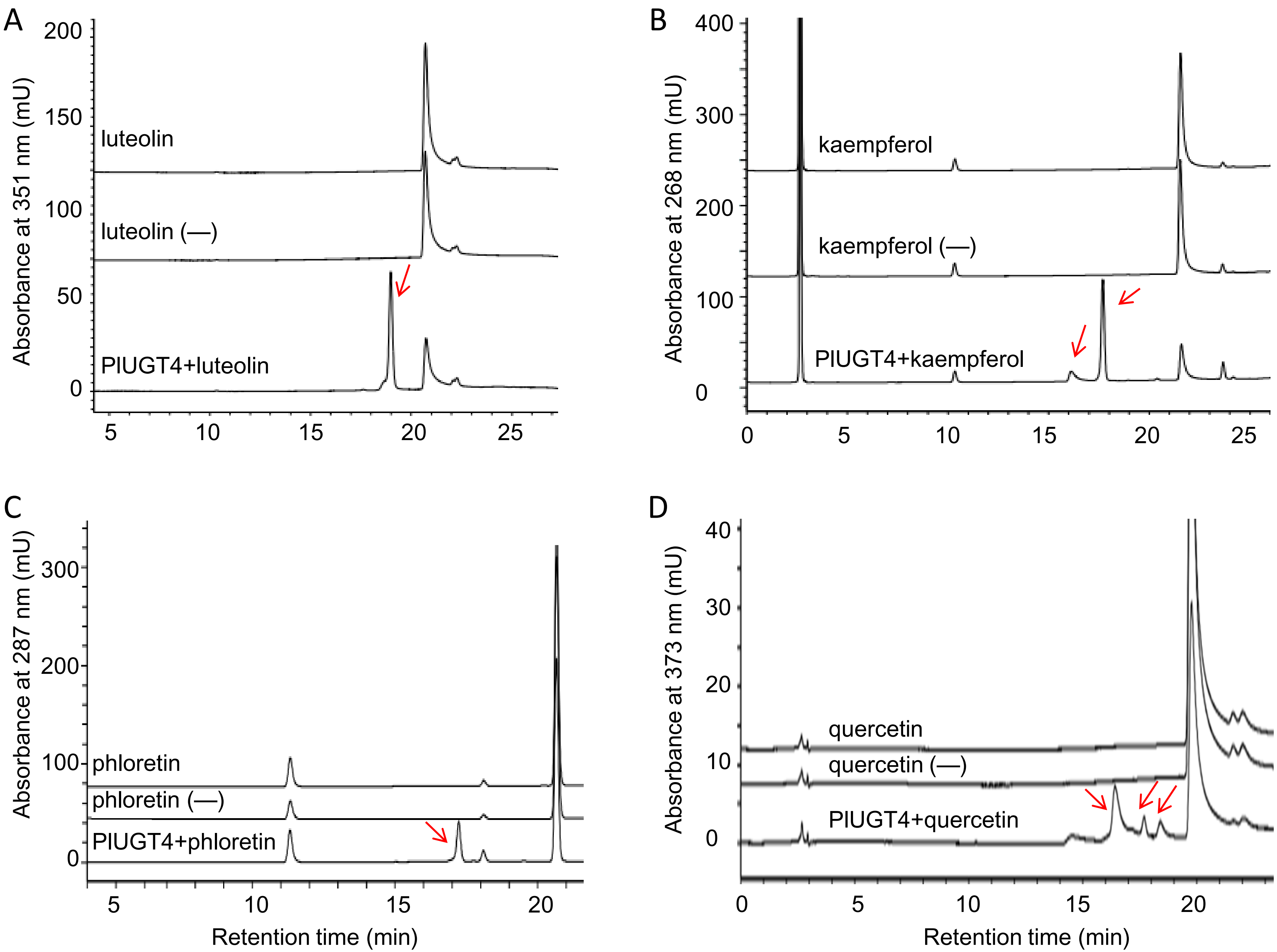
A
Absorbance at 351 nm (mU)
luteolin
luteolin (—)
PlUGT4+luteolin
B
Absorbance at 268 nm (mU)
kaempferol
kaempferol (—)
PlUGT4+kaempferol
C
Absorbance at 287 nm (mU)
phloretin
phloretin (—)
PlUGT4+phloretin
Retention time (min)
D
Absorbance at 373 nm (mU)
quercetin
quercetin (—)
PlUGT4+quercetin
Retention time (min)

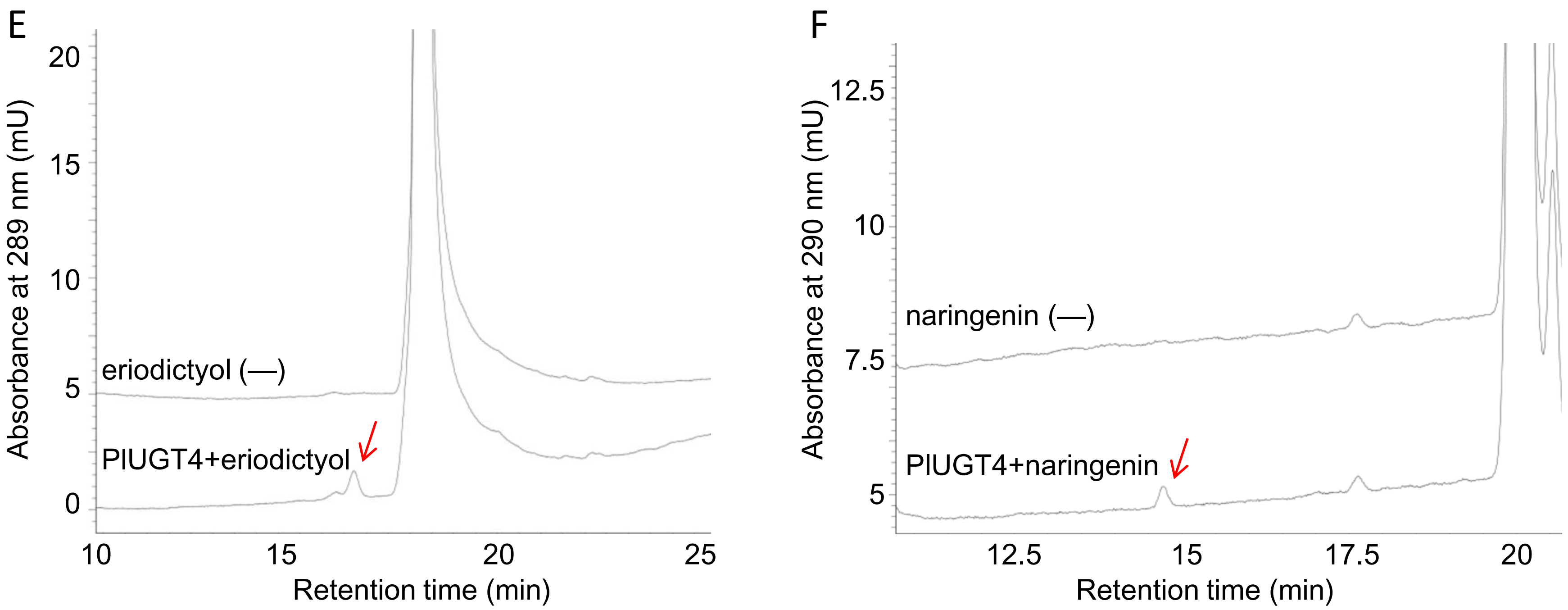

Supplementary Figure S5 HPLC analysis of enzymatic reaction products of PlUGT4 with flavonoid substrates. The UDP-glucose and different flavonoids (A, luteolin; B, kaempferol; C, phloretin; D, quercetin; E, eriodictyol; F, naringenin) were used as the sugar donor and acceptors, respectively. (—) are control assays lacking PlUGT4 protein. The arrows indicate the new peaks (or products) that appear in the presence of PlUGT4 and flavonoid substrates reaction mixtures.
