# Supplementary material for: Identification of Three (Iso)flavonoid Glucosyltransferases From Pueraria lobata
Source: Front Plant Sci. 2019 Jan 25;10:28. doi: 10.3389/fpls.2019.00028 (PMC6362427; doi:10.3389/fpls.2019.00028)
Supplement: Supplementary file 9 [file Image_6.pdf]

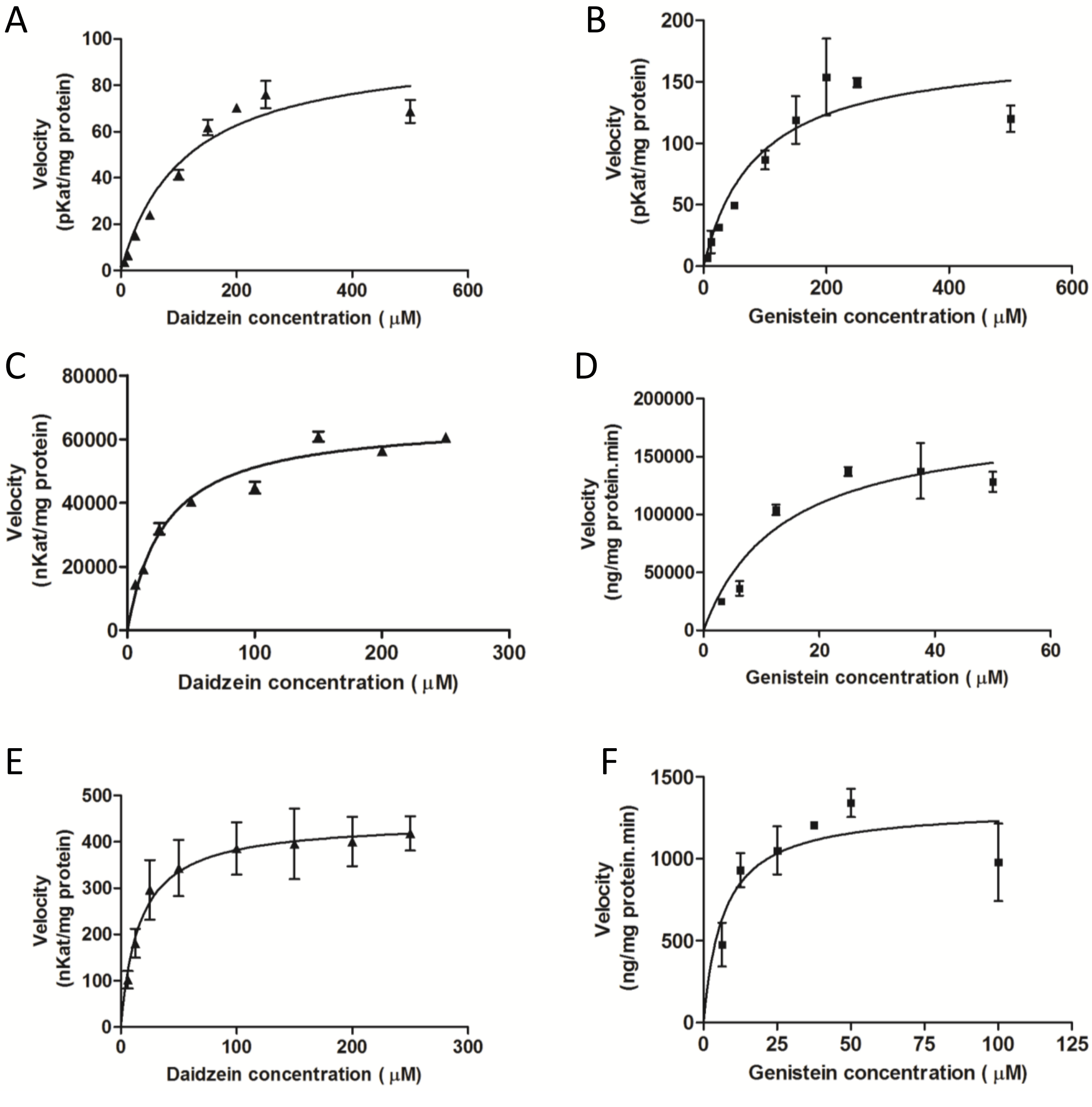

Supplementary Figure S6 The kinetic enzyme curves of the PlUGTs. The recombinant PlUGT4 (A, B), PlUGT15(C, D), and PlUGT57 (E, F) were reacted with different concentrations of daidzein and genistein. Data values were derived from three replicates.
